# Association between self reported snoring, STOP questionnaire and postoperative pulmonary complications in patients submitted to ortophaedic surgery

**DOI:** 10.1186/2049-6958-8-3

**Published:** 2013-01-18

**Authors:** Ahmet Ursavaş, Tahir Güven, Funda Coskun, Ercüment Ege, Aysun Yılmazlar

**Affiliations:** 1 Pulmonary Medicine Department, School of Medicine,, University of Uludag, Bursa, Turkey; 2 Anesthesia Department, School of Medicine, University of Uludag, Bursa, Turkey

**Keywords:** Atelectasis, Obesity, Orthopedic surgery, Sleep apnea, STOP questionnaire

## Abstract

**Background:**

Obstructive sleep apnea (OSA) may increase perioperative complications. The aim of this study was to determine the relationship among postoperative pulmonary complication, snoring and STOP questionnaire in patients with ortophaedic surgery.

**Methods:**

1,406 consecutive records of patients who had undergone elective ortophaedic surgery during the period January 2005-December 2008 were investigated retrospectively. Demographic information, sleep symptoms, STOP questionnaire, comorbidities and outcome data were collected.

**Results:**

There were 289 (20.5%) snorers and 1,117 (79.5%) non-snorers in the study group. There was no significant difference between snorer and non-snorer patients (p > 0.05) in the prevalence of pneumonia and respiratory failure. But in snorer patients the rate of postoperative atelectasis was significantly higher than in non-snorer group (p < 0.0001). The STOP Questionnaire was given to 1,406 patients and 147 (10.4%) out of them were classified at high risk of OSA. There was no significant difference in the prevalence of pneumonia and respiratory failure between low and high risk group (p > 0.05). However, in high risk patients the occurrence of postoperative atelectasis was significantly higher than in low risk group (p < 0.0001).

**Conclusion:**

Postoperative atelectasis was significantly more prevalent in the high risk group according to STOP questionnaire.

## Background

Obstructive sleep apnea (OSA) is a common disorder associated with snoring, witnessed apnea and excessive daytime sleepiness, and significant morbidity and mortality due to cardiovascular events [1-3]. Several population based studies conducted in various geographical regions and ethnic groups demonstrate similar prevalence of OSA, approximately 4% in men and 2% in women [4]. An estimated rate of 82% of men and 92% of women with moderate-to-severe sleep apnea were not diagnosed. Patients with undiagnosed OSA may have increased perioperative complications [5]. Anesthetic agents reduce the tone of pharyngeal musculature, depress ventilation, diminish ventilatory response to carbon dioxide and also abolish arousal from sleep [6]. Snoring is caused by vibration of soft tissues of the upper airways, which in turn is caused by turbulent airflow created by narrowing of one or more cross-sectional areas in the upper airways. Snoring is not necessarily a benign condition, but it can be linked to the serious condition OSA. Habitual snoring (snoring for more than 3 nights a week) has been said to be the best predictor of OSA [7]. Furthermore, simple snoring may be a useful marker for increased cardiovascular risk [8].

The perioperative risk may be reduced by appropriate screening to detect snoring and undiagnosed OSA. Polysomnography is the gold standard method to diagnose OSA. A number of devices utilizable at home and questionnaires are used for screening OSA [9]. The STOP questionnaire is a self-administered screening tool for OSA developed and validated in surgical patients [10].

Perioperative risk factors fell into two general categories: patients- and procedure-related factors. Surgical site, duration of surgery, anesthetic technique, and emergency surgery are procedure-related factors. Orthopaedic surgery has low risk of pulmonary complications except for vertebral surgery [11].

The aim of this study was to determine the relationships among postoperative pulmonary complications, snoring and STOP questionnaire in patients with orthopaedic surgery.

## Methods

### Study population

We retrospectively investigated 1,406 consecutive records of patients aged at least 18 years who had undergone elective orthopaedic surgery from January 2005 to December 2008. Patients with pulmonary infection, and clinically active cardiopulmonary or cerebrovascular disease were excluded from the study. The study was approved by the Institutional Research Ethics Board of Uludag University Medical Hospital, Bursa, Turkey.

### Protocol

Demographic information (age, gender, smoking habits, weight, height, body mass index), American Society of Anesthesiologist (ASA) score, sleep symptoms, STOP questionnaire, comorbidities and outcome data were collected. The STOP questionnaire includes four yes/no questions (Snoring, Tiredness, Observed you stop breathing, blood Pressure). If two or more items are positive, the questionnaire scored as positive. STOP questionnaire should be self-administered, whereas in our study the score was reconstructed retrospectively. ASA score is a system for assessing the fitness of patients before surgery. ASA adopted 6 category physical status classification system. 1- A normal healthy patient, 2- A patient with mild systemic disease, 3- A patient with severe systemic disease, 4- A patient with severe systemic disease that is a constant threat to life, 5- A moribund patient who is not expected to survive without the operation, 6- A declared brain-dead patient whose organs are being removed for donor purposes. Outcome data included postoperative complications such as bronchospasm, pulmonary edema, oxygen desaturation, pneumonia, atelectasis, tachycardia, bradycardia, dysrhythmia, myocardial ischemia confusion, agitation and excessive drowsiness.

### Statistical analysis

Statistical analysis was performed using the SPSS package for Windows, version 13.0. Concordance of normal distribution of all variables was calculated by Shapiro-Wilk test. Student’s *t*-test for independent samples was used for normally distributed continuous variables. The Mann–Whitney *U*-Test was performed for non-normally distributed continuous variables. The results are expressed as mean ± SD. A value of p < 0.05 was considered statistically significant.

## Results

A total of 1,406 patients (784 male, 622 female) aged 17-95 years were evaluated. The overall mean age was 35.4 ± 22.1 years. Seventy two patients were excluded, 30 for questionnaire incompleteness, 12 for pulmonary infection, 18 for active cardiovascular events and 12 for active cerebrovascular diseases.

### Self reported snoring

There were 289 (20.5%) snorers and 1,117 (79.5%) non-snorers in the study group. In snorer patients, age (p < 0.0001), body mass index (BMI: weight/height^2^) (p < 0.0001) and ASA score (p < 0.0001) were significantly higher than in the non-snorer patients. There were 150 (19.2%) snorers among males and 139 (22.3%) among females. There was no significant gender difference between snorers and non-snorer patients (p > 0.05).

In the snorer group, 0.7% pneumonia, 4.2% atelectasis, and 1.4% respiratory failure had been reported during postoperative period, while the incidence was 1%, 0.9%,and 0.5% respectively in the non-snorer group,. Therefore, there was no significant difference in pneumonia and respiratory failure rate between snorer and non-snorer patients (p > 0.05), whereas postoperative atelectasis was significantly higher in snorer than in non-snorer patients (p < 0.0001).

Demographic characteristics and postoperative complications in snorer and non-snorer patients are shown in Table 1.

**Table 1:** Demographic characteristics and postoperative complications in snorer and non-snorer patients

**Variables**	**Snorer (n 289)**	**Non-snorer (n 1,117)**	**p**
Age, year	48.4 ± 19.7	32.1 ± 21.3	< 0.0001
Gender, male	150 (51.9%)	634 (56.8%)	NS
Body mass index, kg/m^2^	27.4 ± 5.6	23.2 ± 5.7	< 0.0001
ASA score	1.6 ± 2.3	1.2 ± 0.57	< 0.0001
Pneumonia	2 (0.7%)	11 (1%)	NS
Atelectasis	12 (4.2%)	10 (0.9%)	< 0.0001
Respiratory failure	4 (1.4%)	6 (0.5%)	NS

NS, Non Significant.

### STOP Questionnaire

The STOP Questionnaire was given to 1,406 patients, 147 (10.4%) out of them classified as being at high risk of OSA. Mean age (p < 0.0001), BMI (p < 0.0001) and ASA score (p < 0.0001) were significantly higher in high risk group when compared to the low risk group (Table 2).

**Table 2:** Demographic characteristics/postoperative complications in patients with high and low risk OSA according to STOP Questionnaire

**Variables**	**High risk**	**Low Risk**	**p**
Age, year	52.7 ± 21.3	33.4 ± 21.2	< 0.0001
Gender, male	81 (55.1%)	541 (43%)	0.005
Body mass index, kg/m^2^	29.4 ± 6.2	23.6 ± 5.7	< 0.0001
ASA score	1.97 ± 3.2	1.29 ± 0.5	< 0.0001
Pneumonia	2 (1.4%)	11 (0.9%)	NS
Atelectasis	9 (13.1%)	13 (1%)	< 0.0001
Respiratory failure	2 (1.4%)	8 (0.6%)	NS

NS, Non Significant.

In high risk group 1.4% pneumonia, 13.1% atelectasis, 0.8% reintubation, and 1.4% respiratory failure had been reported during postoperative period, while in low risk group 0.9% pneumonia, 1% atelectasis, 0.7% reintubation, 0.6% respiratory failure were recorded. Therefore, there was no significant difference in the incidence of pneumonia and respiratory failure between low and high risk groups (p > 0.05). However, in high risk patients the occurrence of postoperative atelectasis was significantly higher than in low risk patients (p < 0.0001).

## Discussion

The main findings of this study were that age, BMI, ASA score and incidence of postoperative atelectasis were significantly higher in snorer and high risk group when compared with non-snorer and low risk group according to STOP questionnaire. To our knowledge, this study is the widest study evaluating the relationships among postoperative pulmonary complications, snoring and results of STOP questionnaire in patients with orthopaedic surgery.

OSA is a prevalent condition in general population. There are several factors which increase the rate of perioperative complications in patients with OSA [5]. Anesthetics and analgesics increase upper airway resistance by interfering with the function of upper airway muscles and also affect respiratory control. Furthermore, trauma from airway manipulation, drugs and pain all can affect sleep architecture and regulation of upper airway muscles in the postoperative period [12]. The most common perioperative complications in OSA patients are exacerbation of obstructive apnea due to upper airway collapse, hypoxemia, hypercapnia, difficulties of airway management, postoperative infection, atelectasis, encephalopathy, cardiac ischemia and arrhythmias [13-15].

There has been little study about perioperative complications in OSA patients. Most studies in the literature are retrospective, observational or case reports [5]. Randomized controlled trials may be difficult to perform due to ethical consideration. The risk of respiratory complications such as hypoxemia, airway obstruction and hypoventilation was 1.3% in surgical patients given general anesthesia in one study, and although OSA risk was not investigated, the use of opioids was associated with greater perioperative pulmonary complications [16].

Surgical site and anesthetic technique are important factors to predict perioperative pulmonary complications. There are a number of reports of perioperative risk in OSA patients undergone upper airway surgery such as uvulopalatopharyngoplasty (UPPP) and tonsillectomy. Pang [17] reported 118 treated patients and 152 surgical procedures: overall peri- and post-operative complication rate was 13.8%, all patients were treated accordingly and recovered well, with no mortality. The author [17] concluded that patients with severe OSA (apnoea-hypopnoea index > 60 and lowest oxygen saturation < 80%) are at higher risk of post-operative oxygen desaturation.

The effect of OSA in other surgical procedures such as thoracic, abdominal or orthopaedic was less investigated. Kaw et al. [15] looked at 25,587 patients who underwent cardiac surgery. Out of these, 37 patients were also identified as having OSA. A higher incidence of encephalopathy, postoperative infection and increased length of stay in ICU were noted in the group with OSA after cardiac surgery. The difference in the rates of infection was mostly due to the presence of mediastinitis. Differences in the rate of reintubation, tube time, and overall postoperative morbidity were not statistically significant. There is only one study similar to our investigation which related to OSA and orthopaedic surgery. Gupta et al. [18] carried out a retrospective case control study of OSA patients undergoing hip or knee replacement surgery. They found an increased incidence of postoperative complications (2.5 times), an elevated intensive care unit need, and longer hospital stays in OSA patients *versus* controls. In our study snoring and high risk OSA according to STOP questionnaire were associated with increased incidence of postoperative atelectasis.

There are several confounding factors that can have effect on the relationships between OSA and postoperative complications. Obesity and anesthetic-analgesic drugs are the most important of these factors. Respiratory and cardiac physiology, pharmacokinetics of drugs, positioning, regional anesthetic techniques, monitoring, and postoperative care are all profoundly affected by obesity [19]. OSA is also more prevalent in obese individuals. Population based cohort studies revealed that obesity is an independent risk factor for OSA. Sixty percent of OSA patients are obese [20]. Some of the postoperative complications are also directly related to obesity without apnea in obese OSA patients. Obesity is associated with reduction in functional residual capacity (FRC), expiratory reserve volume and total lung capacity. FRC may be reduced in the upright obese patient to the extent that it falls within the range of the closing capacity with subsequent small airway closure, atelectasis, ventilation-perfusion mismatch, right to left shunting and arterial hypoxaemia [21]. In our study, in snorer and high risk group BMI was significantly higher than in non-snorer and low risk group. Atelectasis is more prevalent in snorer and high risk group and this may be related to obesity.

Some potential limitations of this study should be considered, of course. Firstly, our study is retrospective and STOP questionnaire was based on self subjective reports. Furthermore STOP questionnaire should be self-administered, whereas in our study the score was reconstructed retrospectively. Secondly, there is no particular study which evaluated the reliability and validity of STOP questionnaire in our population. However, the same problems exist in other similar studies. Thirdly, we did not perform any sleep study such as oximetry or polysomnography.

## Conclusions

In conclusion, although there were no significant differences in pneumonia and respiratory failure rate between high and low risk group, atelectasis was significantly more prevalent in the group at high risk according to STOP questionnaire.

## Competing interests

The authors declare that they have no competing interests.
